# Complete Genome Sequence of Bacillus cereus Strain HT18, Isolated from Forest Soil

**DOI:** 10.1128/mra.01106-21

**Published:** 2022-03-07

**Authors:** Minami Matsunaka, Nguyen Cong Thanh, Tatsuya Uedoi, Takashi Iida, Yasuhiro Fujino, Taketo Ohmori, Yasuaki Hiromasa, Toshihisa Ohshima, Katsumi Doi

**Affiliations:** a Microbial Genetic Division, Institute of Genetic Resources, Faculty of Agriculture, Kyushu University, Fukuoka, Japan; b Plant Protection Research Institute, Hanoi, Vietnam; c Department of Biomedical Engineering, Faculty of Engineering, Osaka Institute of Technology, Osaka, Japan; d Attached Promotive Center for International Education and Research of Agriculture, Faculty of Agriculture, Kyushu University, Fukuoka, Japan; University of Rochester School of Medicine and Dentistry

## Abstract

The genome sequence of Bacillus cereus strain HT18, isolated from forest soil, was 5,333,415 bp long. The genome included 5,825 putative coding sequences and 35.2% GC content; the strain had 5 plasmids. Average nucleotide identity based on BLAST+ (ANIb) and digital DNA-DNA hybridization (dDDH) results showed that HT18 was 98.78% and 90.70% homologous, respectively, to B. cereus ATCC 14579^T^.

## ANNOUNCEMENT

The Bacillus cereus group (phylum *Firmicutes*) comprises Gram-positive, spore-forming, facultative, anaerobic, rod-shaped bacteria with low-GC-content genomes (1). It includes eight closely related species with high genomic homology and 16S rRNA gene sequence similarity—B. anthracis, B. cereus, B. cytotoxicus, B. mycoides, B. pseudomycoides, B. thuringiensis, B. toyonensis, and B. weihenstephanensis (2). Phenotypic features, such as motility and hemolysis, used to classify species within this group can differ within and among species, leading to the use of the average nucleotide identity based on BLAST+ (ANIb) and digital DNA-DNA hybridization (dDDH) as classification indices (3, 4).

Strain HT18 was isolated from forest soil in Hashimoto, Wakayama, Japan. The soil samples were flooded, filtered, and incubated overnight at 37°C on LB agar (Nacalai Tesque). After colony isolation, the cells were cultured in LB broth at 37°C for 24 h. Then, genomic DNA was isolated using Marmur’s method (5).

Sequencing was a combination of short and long reads. Short-read sequencing libraries were constructed using the NEBNext Ultra II FS DNA library prep kit (New England Biolabs [NEB]) and decoded on a MiSeq instrument (Illumina). A total of 1,488,012 reads with 431,000,004 bases were decoded with an average insert size of 641 bp and spot length of 602 bp with 2 × 300-bp paired ends. Low-quality bases (Q scores of <15) were trimmed, and short reads (<25 bp) were removed using Platanus trim version 1.0.7 (http://platanus.bio.titech.ac.jp/pltanus_trim) (6). Long-read sequence libraries were constructed using the rapid barcoding kit (SQK-RBK004), and sequencing was performed on a MinION device (Oxford Nanopore Technology [ONT]) using an R9.4.1 flow cell. No size selection was performed before library preparation. A total of 48,894 reads with 455,539,372 bases were decoded using Guppy version 4.0.15. The recovered data with NanoFilter technology (quality, ≥10; length, ≥2 kb; head crop, 100 bp) revealed 23,033 ONT reads with a mean read length of 17,050 bp and an *N*_50_ value of 27,780 bp. *De novo* assembly was performed using Unicycler version 0.4.8 (https://github.com/rrwick/Unicycler) (7). The genome annotation and rotation of the chromosome to bring *dnaA* first was performed with DFAST version 1.4.0 (https://dfast.ddbj.nig.ac.jp) (8). ANIb and dDDH values were calculated from the JSpeciesWS online service version 3.8.5 (http://jspecies.ribohost.com/jspeciesws/) and the Genome-to-Genome Distance Calculator version 3.0 (http://ggdc.dsmz.de/ggdc.php) (9) using the reported genome sequences of type strains of the B. cereus group. Default parameters were used for all software, unless specified.

One contig of the assembled genome sequence was 5,333,415 bp long (35.2% GC content) with a sequence depth of approximately 80. A total of 5,825 coding regions, 107 tRNAs, 42 rRNAs, and 3 CRISPR regions were annotated. Five plasmid DNA sequences were also assembled (Table 1).

**TABLE 1:** Characteristics of the genome of Bacillus cereus strain HT18

Genetic element	Assembly size (bp)	G+C content (%)	No. of CDSs^*a*^	No. of rRNAs	No. of tRNAs	Avg read depth (×)	Accession no.
Chromosome	5,333,415	35.2	5,488	42	107	75.8	AP024504
Plasmids							
pHT1	358,139	33.4	355	0	0	130.4	AP024505
pHT2	9,518	31.6	12	0	0	351.7	AP024506
pHT3	9,190	31.4	10	0	0	537.4	AP024507
pHT4	7,215	32.1	7	0	0	687.5	AP024508
pHT5	3,166	33.1	3	0	0	476.0	AP024509

a CDS, coding DNA sequence.

Based on the ANIb (%) and dDDH (%) results, strain HT18 was 99.9% and 100% homologous to both B. cereus strains WPySW2 and AFA01, and 99.9% and 99.9% homologous to the foodborne pathogen FORC021 (10) (Table S1, posted at https://figshare.com/articles/dataset/suppl_Table_pdf/17111096). Although strain HT18 showed high homology to B. thuringiensis serovar Berliner ATCC 10792^T^, it was closely related to B. cereus ATCC 14579^T^ when it was compared to reference strains of the B. cereus group. Therefore, strain HT18 was identified as a B. cereus strain.

### Data availability.

The genome sequence and annotation data for strain HT18 were deposited in DDBJ/GenBank under BioProject number PRJDB11181, BioSample number SAMD00278679, DRA number DRA011610, accession numbers AP024504 to AP024509, and SRA numbers DRX266213 (Illumina) and DRX266214 (ONT).
